# Understanding Synergistic Toxicity of Terpenes as Insecticides: Contribution of Metabolic Detoxification in *Musca domestica*

**DOI:** 10.3389/fpls.2018.01579

**Published:** 2018-10-30

**Authors:** Esteban Scalerandi, Guillermo A. Flores, Marcela Palacio, Maria Teresa Defagó, María Cecilia Carpinella, Graciela Valladares, Alberto Bertoni, Sara María Palacios

**Affiliations:** ^1^Centro de Investigaciones Entomológicas de Córdoba, IMBIV, CONICET-UNC - Universidad Nacional de Córdoba, Córdoba, Argentina; ^2^IMBIV, CONICET-UNC - Universidad Nacional de Córdoba, Córdoba, Argentina; ^3^Laboratorio de Química Fina y Productos Naturales, IRNASUS-CONICET-Universidad Católica de Córdoba, Córdoba, Argentina

**Keywords:** *Musca domestica*, fumigant toxicity, 1, 8-cineole, citronellal, linalool, (*R*)-pulegone, synergism

## Abstract

Essential oils, which are mixtures of terpenes, frequently show stronger insecticide activity, i.e., lower lethal dose 50 (LC_50_), than their most abundant terpenes. Synergy between terpenes provides a plausible explanation, but its demonstration has been elusive. In the present work, we look for an alternative explanation, by considering the influence of insect metabolic detoxification. Basically, we propose a model (metabolic model, MM) in which the LC_50_ of the major terpene in a mixture is expected to include a fraction that is detoxified by the insect, whereas a minor terpene would act unimpeded, showing a lower LC_50_ than when acting alone. In order to test this idea, we analyzed the effects of inhibiting the cytochrome P450 detoxification system with piperonyl butoxide (PBO), on the lethal concentration of terpenes as fumigants against *Musca domestica*. We found that, within a group of 10 terpenes [linalool, citronellal, (*R*)-α-pinene, 1,8-cineole, γ-terpinene, limonene, α-terpinene, (*S*)-β-pinene, thymol and (*R*)-pulegone], seven showed the LC_50_PBO (the lethal concentration for PBO-treated flies) between 1.7 and 12.4 times lower than the corresponding LC_50_ when P450 was not inhibited. Only in one case, that of (*R*)-pulegone, was the LC_50_PBO greater than the LC_50_, while two terpenes [(*S*)-β-pinene and thymol] showed no changes in toxicity. The increased activity of most terpenes (particularly linalool and citronellal) in PBO-treated flies supports our hypothesis that normally the LC_50_ includes a fraction of inactive compound, due to detoxification. Having previously determined that *M. domestica* preferentially oxidizes the most abundant terpene in a mixture, while terpenes in smaller proportions are poorly or not detoxified by the P450 system, we assessed whether the toxicity of minority terpenes in a mixture is similar to their activity under P450 inhibition. We chose suitable binary combinations in such a way that one terpene (in greater proportion) should be the target of P450 while the other (in smaller proportion) should intoxicate the fly with LC_50_PBO or similar. Combinations of 1,8-cineole-citronellal, 1,8-cineole-linalool, linalool-citronellal, (*R*)-pulegone-linalool, (*R*)-pulegone-1,8-cineole and (*R*)-pulegone-citronellal were assayed against *M. domestica*, and the LC_50_ of each mixture was determined and compared to values predicted by MM (considering the LC_50_PBO for minor component) or by the classical approach (LC_50_ for both components). The MM showed the best fit to the data, suggesting additive rather than synergistic effects, except for the combination of (*R*)-pulegone-citronellal that was clearly synergistic. Thus, the experimental data indicate that the insect preferentially oxidizes the major component in a mixture, while the terpene in lesser proportion acts as a toxicant, with higher toxicity than when it was assayed alone. These findings contribute to a deeper understanding of the higher toxicity of essential oils compared to their component terpenes and provide important information for the design of effective insecticides based on essential oils or terpenes.

## Introduction

Terpenes are secondary metabolites widely distributed in the plant kingdom. Plants produce mixtures of terpenes, which are easily isolated by hydrodistillation as oils, generally known as essential oils. The oil composition is characteristic of each plant species, with normally 1–3 terpenes as major components and many as minor components (Raut and Karuppayil, 2014). Terpenes are often volatile and commonly used as fragrances, flavoring agents or in medicine (e.g., aromatherapy) (Ali et al., 2015). In nature, they play an important role in plant defense against a variety of organisms (Bakkali et al., 2008; Pavela and Benelli, 2016). In particular, their demonstrated toxicity against many insect pests makes terpenes excellent candidates for the development of eco-friendly pesticides (Isman, 2000; Miresmailli and Isman, 2014). Thus, various essential oils and their terpenes have proven contact and fumigant toxic effects against mosquitoes (Maheswaran and Ignacimuthu, 2012; Pavela, 2015; Govindarajan et al., 2016), house flies (Rossi et al., 2012; Rossi and Palacios, 2013, Rossi and Palacios, 2015; Benelli et al., 2017, 2018a,b; Zhang et al., 2017), lepidoptera larvae (Pavela, 2014), stored grain pests (Rajendran and Sriranjini, 2008; Herrera et al., 2015) and many other insect pests.

The biological activity of essential oils depends not only on their qualitative composition, but also on the quantitative ratio of their components (Bekele and Hassanali, 2001; Pavela, 2008). These oils have been repeatedly found to be more active, i.e., showing lower lethal concentration (LC_50_), than the most active of their component terpenes. For example, in fumigation experiments, the essential oil of *Minthostachys verticillata* was toxic to *Musca domestica*, but it took 3.4 times more of its main component, (*R*)-pulegone, to obtain the same toxicity (Rossi et al., 2012). Similarly, fumigant thyme oil was 2.7 times more toxic than thymol, its major terpene (Tak et al., 2016), and lemongrass essential oil showed a lower topical LC_50_ against *Trichoplusia ni* larvae than its most abundant terpene, citral (Tak et al., 2016). As yet, there is no conclusive explanation of this discrepancy. Some studies have found synergy between the terpenes that compose an essential oil (Pavela, 2014), but others have not or have only partially demonstrated synergy (Passreiter et al., 2004; Tak and Isman, 2016). Recent studies have also shown that enhanced penetration of a given terpene by the presence of another less toxic terpene may result in a synergic combination (Tak and Isman, 2017a,b).

In this context, we would like to highlight a factor overlooked until now: the influence of metabolic detoxification by the insects, which may underlie the greater toxicity of full essential oils relative to their main components. Insects have evolved alongside plant defenses like natural terpenes, so they have developed mechanisms to deal with them to some extent (Rosenthal and Berenbaum, 2012). There is evidence that insect metabolism can alter the actual concentration of these toxicants, as demonstrated for the essential oils from *M. verticillata*, eucalyptus (*Eucalyptus cinerea*) and orange (*Citrus sinensis*) as fumigants against *M. domestica* (Rossi et al., 2012; Rossi and Palacios, 2013, Rossi and Palacios, 2015). In these cases, the essential oils were more active than the most abundant (and most toxic) terpene, and it was shown that *M. domestica* absorbed most of the component terpenes, proportionally to their relative contribution to the essential oils. The metabolization of the major terpene by cytochrome P450 was also demonstrated in each case, by using piperonyl butoxide (PBO) to inhibit the oxidizing system (Jones, 1998). In other words, the insect absorbs most of the terpenes offered, but preferentially metabolizes the terpene with the highest concentration in the mixture. The metabolic process could yield a product even more toxic to the insect than the original terpene, thus enhancing its toxicity (Rossi et al., 2012), but would more often diminish the toxicity of that terpene (Rossi and Palacios, 2013, Rossi and Palacios, 2015).

The toxicity of a given terpene is usually described in terms of the observed LC_50_ which, considering the metabolization discussed above, could actually be conceived as LC_50_ = A + B, where A accounts for the dose fraction necessary to reach the action site and produce the effect, and B represents the dose fraction lost by the insect metabolic response. However, the contribution of A and B to each terpene’s LC_50_ value is not known. An approximation can be provided by inhibiting the metabolic pathway, for example using PBO as a P450 inhibitor. In this case, the A-value would approximate what we will call LC_50_PBO, i.e., the LC_50_ observed when applying terpene on insects previously treated with PBO, so that the metabolic response is inhibited and therefore B = 0 (at least for terpenes detoxified by this via).

Loss of essential oil due to insect metabolism (B fraction) in the above studies represented, almost exclusively, detoxification of the major component (Rossi et al., 2012; Rossi and Palacios, 2013, Rossi and Palacios, 2015). It is then possible that while the most abundant terpene is being dealt with, other terpenes could intoxicate the insect. In that case, the insecticidal potency of a minor terpene LC_50_ = A + B will be composed mostly of portion A, as this terpene is not or hardly detoxified (B ≈ 0). With this idea in mind, we propose a novel way of analysis for the study of synergistic effects of terpenes on insects, which incorporates the insect metabolic response. In what we have called the Metabolic Model (MM), the major terpene in a mixture is expected to intoxicate the target insect with a typical LC_50_ (LC_50_ = A + B) whereas the minority terpene would do the same with an LC_50_PBO (LC_50_ = A). Instead, the classical approach (which we have called Classical Model, CM) expects toxicity of a given terpene to be represented by its LC_50_.

In order to test our model, we used *M. domestica* L. (Diptera: Muscidae) as a target insect and a group of 10 terpenes which we previously proved to be effective fumigants against this pest. We determined the LC_50_ of these terpenes as well as their LC_50_PBO, which we used as a close estimation of LC_50_ = A. Then, we bioassayed mixtures of these terpenes in varying proportions, and the observed LC_50_ values were compared with the values predicted for each mixture according to both models.

## Materials and Methods

### Chemicals

1,8-Cineole, citronellal, citronellic acid, citronellol, linalool, (*R*)-limonene, (*R*)-α-pinene, (*S*)-β-pinene, (*R*)-pulegone, α-terpinene, γ-terpinene, thymol and PBO used in the bioassays or as chromatographic standards were purchased from Sigma-Aldrich (St. Louis, MO, United States). HPLC grade acetone was purchased from Merck (Darmstadt, Germany).

### House Flies

The colonies of *M. domestica* originated from adults collected from the experimental farm of the Universidad Católica of Córdoba, in Córdoba, Argentina in February 2016, using a sweep net. The flies were reared in entomological cages (30 cm × 30 cm × 30 cm), under the following conditions: 26 ± 1°C, 70% humidity and 12:12 light–dark cycle, with a diet of powdered milk and water. A mixture prepared with bran (90 g), baker’s yeast (0.5 g) and milk powder (10 g) provided oviposition sites.

### Bioassay

The bioassay against *M. domestica* was performed as previously reported (Palacios et al., 2009a,b). Briefly, ten 4–5-day-old adult houseflies, both sexes, were placed in a glass jar (1.2 L) fitted with a screw cap which held a 7-cm length of cotton yarn suspended from the center of its inner surface. Dosages of a given terpene or a mixture of two terpenes (dissolved in 20 μL acetone) were applied to the yarn. The jars were sealed tightly and kept in a room at 26 ± 1°C for 30 min. Each test was replicated three times. The control vessel had only acetone on the cotton yarn. Mortality in each group was assessed after 30 min of exposure by softly stimulating each fly with the tip of a pen. Flies that did not respond were considered dead. The mortality thus determined was used to calculate the LC_50_ of the corresponding compound. Mixtures were prepared from pure terpene in the desired proportions, dissolved in acetone, and assayed as described above.

### Determination of LC_50_PBO

Each terpene was assayed in combination with PBO, according to the method previously reported (Rossi et al., 2012). A fumigation bioassay of each terpene (in doses from 0.5 mg/L to 18 mg/L) was then performed with the PBO-treated flies, as described above. Control groups with insects also topicated with PBO received only acetone. The mortality values were used to determine LC_50_PBO.

### Determination of Predicted LC_50_ According to CM and MM

The formula used to calculate LC_50_ CM or MM is presented in Eq. 1 (Tallarida and Raffa, 1995):

(1) $$\begin{array}{l} {\text{LC}}_{50}(\text{mix})={\text{LC}}_{50}{\text{T}}_{\text{I}}/f{\text{T}}_{\text{I}}(1-{\text{LC}}_{50}{\text{T}}_{\text{I}}/{\text{LC}}_{50}{\text{T}}_{\text{II}})\\ \;+({\text{LC}}_{50}{\text{T}}_{\text{I}}/{\text{LC}}_{50}{\text{T}}_{\text{II}}) \end{array}$$

where *f*T_I_ is the molar fraction of terpene I and *T*_II_ represents another terpene in the mixture. LC_50_ CM were calculated using the LC_50_ of Table 1 for both terpenes in the mixture and their corresponding proportions in the mixture. LC_50_ MM were calculated using the LC_50_ (Table 1) for the major terpene and the LC_50_PBO (Table 1) for the minority terpene with their respective proportions.

**Table 1:** Fumigant toxicity of selected terpenes with or without PBO against *Musca domestica* adults.

Terpene	LC_50_^a^	LC_50_PBO^b^	Ratio LC_50_/LC_50_PBO
Linalool	13.6 (11.8–15.8)	1.1 (0.4–1.9)	12.4
Citronellal	8.1 (2.8–23.5)	0.9 (0.7–1.2)	9.0
(*R*)-α-pinene	12.1 (9.5–15.5)	4.9 (2.5–8.5)	2.5
1,8-cineole	3.3 (1.1–10.4)	1.6 (0.9–3.1)^c^	2.1
γ-terpinene	4.0 (2.5–10.9)	2.1 (1.5–2.9)	1.9
(*R*)-limonene	6.2 (1.7–23)	3.6 (1.4–9.8)^d^	1.7
α-terpinene	6.2 (4.8–13.7)	3.6 (3.0–4.2)	1.7
(*S*)-β-pinene	6.4 (2.4–17.4)	5.9 (5.1–6.6)	1.1
Thymol	13 (2.4–68.7)	12.8 (9.8–18.1)	1.0
(*R*)-pulegone	1.7 (0.6–5.0)	4.4 (1.1–18.2)^e^	0.4

*^a^Taken from (Palacios et al., 2009a,b). ^b^Musca domestica adults were previously topicated with 10 μgPBO/fly. ^c^Taken from Rossi and Palacios (2013). ^d^Taken from Rossi and Palacios (2015). ^e^Taken from Rossi et al. (2012). LC_50_ and LC_50_PBO values were calculated from 4 to 5 concentrations, three replicates each. Values in brackets are the 95% confidence interval.*

### Determination of Combination Index and Dose-Reduction Index

The combination index (CI) and the dose reduction index (DRI) were calculated by Compusyn software (Chou, 2006) using the LC_50_ data for the corresponding case.

CI values of <1 indicate synergy, >1 indicate antagonism and = 1 indicates additive effect.

The dose-reduction index (DRI) provides a measure of how much the dose of each terpene in a synergistic combination may be reduced at a given effect level, (i.e., at 50% mortality) compared with the doses of each terpene alone (Chou, 2006).

### Determination of Metabolites Produced by House Flies

After a fumigation bioassay with citronellal (with *n* = 50), the dead flies (∼250 mg) were collected in a vial (10 mL volume) with a septum. The vial was then placed in a bath at 60°C for 15 min. The terpenes desorbed from the *M. domestica* to the headspace of the vial were captured using a SPME microfiber (Supelco, Bellefonte, PA, United States; with polydimethylsiloxane, thickness 30 μm, length 1 cm). The microfiber was injected in a Perkin-Elmer Clarus 600 gas chromatograph coupled with an ion trap mass detector (GC-MS) for terpene and metabolite identification. An ELITE 5-ms capillary column (60 m × 0.25 mm i.d. and 0.25 mm coating thickness) was used for the separation of components. The chromatographic conditions were: injector at 200°C; oven temperature programming 80°C (5 min) to 240°C at 5°C/min; constant detector temperature of 240°C. Helium was used as the carrier gas (flow rate 1.5 mL/min). Compounds were identified by comparing their mass spectra with available libraries (NIST) and confirmed by co-injection of pure standards (Sigma, United States).

Experiments with (*R*)-pulegone/citronellal combinations were analyzed using the same methodology as described above.

### Statistical Analysis

The mean mortality data of the three replicates per dose (4–5 doses per terpene or mixtures) were used to determine the LC_50_ and LC_50_PBO. Probit analysis (POLO-PLUS software, LeOra Software, United States) was used to analyze the dose–mortality response. GraphPad Prism 5 was used to draw the isobologram graphs (Tang et al., 2002).

## Results and Discussion

The LC_50_ values of the studied terpenes on *M. domestica* adults, with and without previous topication with PBO (Table 1), show that nearly all of the assayed terpenes were more toxic (lower LC_50_PBO) when P450 were inhibited, suggesting that they are actually detoxified by this oxidant complex. Only (*R*)-pulegone was less toxic when the detoxificant mechanism was inhibited, which reflects the high toxicity of the oxidized (*R*)-pulegone metabolite, menthofuran, to *M. domestica* adults (Rossi et al., 2012). The largest LC_50_/LC_50_PBO ratio was observed for linalool and citronellal, which became highly toxic for this insect under P450 inhibition, despite being some of the least effective terpenes in normal conditions, thus suggesting a very efficient fly detoxification for these compounds. Both compounds have easily oxidizable functional groups, which crucially allow the necessary structural changes for easier detoxification of these substances.

Most studies on the toxicity of terpene mixtures, following the classical model, tend to combine the most active terpene in 50:50 proportion (Hummelbrunner and Isman, 2001), or mix them in their LC_25_ to demonstrate synergism (Pavela, 2015; Kanda et al., 2017). However, if the minor terpene were to act with an LC_50_PBO (=A), more interesting results could be obtained by testing different proportions in order to allow a major terpene to be a target of the metabolic process, while a minor terpene intoxicates the insect (metabolic model). The lists of best possible combinations arising from these two approaches differ appreciably (Table 2), with only one combination [(*R*)-pulegone/1,8-cineole] being common to both lists.

**Table 2:** Predicted best combinations according to the Classical Model (CM) and the Metabolic Model (MM).

Classical Model (CM)		Metabolic model (MM)	
Terpene I (any proportion)	Terpene II (any proportion)	Terpene I (major proportion)	Terpene II (minor proportion)
(*R*)-pulegone	1,8-cineole	(*R*)-pulegone	Citronellal
(*R*)-pulegone	γ-terpinene	(*R*)-pulegone	Linalool
(*R*)-pulegone	(*R*)-limonene	(*R*)-pulegone	1,8-cineole
1,8-cineole	γ -terpinene	1,8-cineole	Citronellal
1,8-cineole	Limonene	1,8-cineole	Linalool

When we assayed some of the best combinations predicted by the MM (Table 2) and compared the observed LC_50_ with values predicted by each model, we found that MM showed a better fit to observed values than CM. In fact, the observed LC_50_ values were always lower than those predicted by CM, but were quite similar to those calculated following MM (Table 3: entries 4, 5, 6), thus suggesting additive effects between the terpenes if the metabolic contribution is discounted. However, observed LC_50_ values for the mixtures with (*R*)-pulegone (Table 3: entries 1, 2, 3) were lower even than predictions from MM. So, we further explored both types of situation by testing mixtures with a variety of proportions, as discussed below.

**Table 3:** Fumigant toxicity of selected mixtures of terpenes: observed and predicted LC_50_, according to the CM and MM, against *Musca domestica* adults.

Entry	Combination (%)		Observed LC_50_ mg/L	Predicted (CM) LC_50_ mg/L	Predicted (MM) LC_50_ mg/L
1	(*R*)-pulegone (70)	Citronellal (30)	0.26 (0.19–0.34)	2.48	1.29
2	(*R*)-pulegone (70)	Linalool (30)	0.79 (0.58–1.03)	2.30	1.46
3	(*R*)-pulegone (70)	1,8-cineole (30)	0.78 (0.52–1.12)	1.99	1.67
4	1,8-cineole (70)	Citronellal (30)	1.99 (1.40–2.50)	4.01	1.91
5	1,8-cineole (70)	Linalool (30)	1.98 (1.66–2.36)	4.27	2.06
6	Citronellal (70)	Linalool (30)	3.37 (2.16–5.41)	9.22	3.86

*Observed values are accompanied by their 95% confidence interval, in brackets.*

### Additive Effect of Terpenes

**FIGURE 1:**
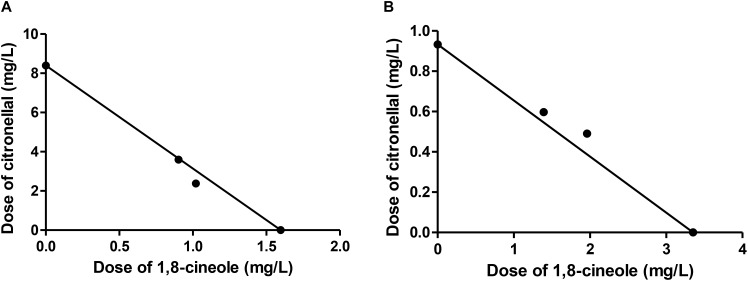
Isobologram corresponding to the 1,8-cineole-citronellal mixtures as fumigants against *Musca domestica*. The metabolic model (MM) was considered with **(A)** citronellal or **(B)** 1,8-cineole as substrate of P450. The isobolograms suggest an additive effect in each case.

In all combinations of 1,8-cineole and citronellal against *M. domestica*, observed LC_50_ values were very close to those predicted by MM (Table 4). When these data were analyzed following the theoretical basis of drug combination studies proposed by Chou (Chou, 2006), the term combination index (CI) for quantification of interactions between two compounds showed values close to one, thus suggesting an additive interaction. The isobologram analysis also corroborated the additive effect (Figure 1). These results match perfectly with our model, based on the contention that the major component of the mixture is metabolized by P450 but the minor component is poorly or not detoxified by this enzyme complex. These findings highlight the need to consider the insect metabolic response when studying toxic compounds, and suggest that the apparent synergy in the CM analysis could simply reflect unawareness of losses due to detoxification by the insect.

**Table 4:** Fumigant toxicity of 1,8-cineole and citronellal mixtures against *Musca domestica* adults.

Entry	Combination (% w/w)	Observed^a^ LC_50_ mg/L	Predicted (MM)^b^ LC_50_ mg/L	CI
1	1,8-cineole/citronellal (80:20)	2.45 (2.22–2.69)	2.23	1.17
2	1,8-cineole/citronellal (70:30)	1.99 (1.41–2.50)	1.91	1.2
3	1,8-cineole/citronellal (30:70)	3.37 (2.68–3.95)	3.65	0.92
4	1,8-cineole/citronellal (20:80)	4.50 (3.84–5.21)	4.46	0.99

*^a^Values in brackets are the 95% confidence interval. ^b^For calculation of LC_50_ (MM), values used for 1,8-cineole were 3.3 and 1.5 mg/L for the 1, 2 and for 3, 4 entries, respectively. Values used for citronellal were 0.9 and 8.1 mg/ml for 1, 2 and for 3, 4 entries, respectively. CI, combination index (Chou, 2006).*

**FIGURE 2:**
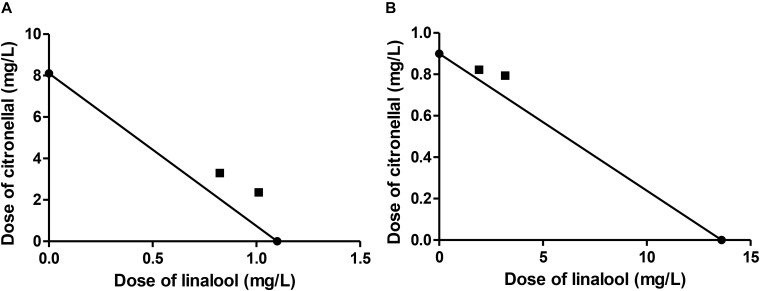
Isobologram corresponding to the linalool-citronellal mixtures as fumigants against *M. domestica*. The MM was considered with **(A)** citronellal or **(B)** linalool as substrate of P450. The isobolograms suggest an additive effect in each case.

The combinations of linalool and citronellal assayed against *M. domestica* produced observed-LC_50_ values very close to those predicted from MM (Table 5). Moreover, the CI values obtained for these combinations were close to one (Table 5 and Figure 2), suggesting an additive interaction and supporting our hypothesis that the major component represented the target of the oxidative system while the minor terpene was less or not detoxified. These and the above results allow us to postulate that not all mixtures that are considered synergistic in the literature are necessarily so, but might actually turn out to be additive, if the MM is considered.

**Table 5:** Fumigant toxicity of linalool and citronellal mixtures against *Musca domestica* adults.

Entry	Combination (% w/w)	Observed^a^ LC_50_ mg/L	Predicted (MM)^b^ LC_50_ mg/L	CI
1	Linalool/citronellal (80:20)	3.97 (3.04–5.41)	3.77	1.1
2	Linalool/citronellal (70:30)	2.74 (2.18–3.37)	2.77	1.0
3	Linalool/citronellal (30:70)	3.37 (2.16–5.41)	3.86	1.2
4	Linalool/citronellal (20:80)	4.12 (3.12–5.50)	4.67	1.1

*^a^Values in brackets are the 95% confidence interval. ^b^For calculation of LC_50_ (MM), values used for linalool were 13.6 and 1.1 mg/L for the 1, 2 and for 3, 4 entries, respectively. Values used for citronellal were 0.9 and 8.1 mg/ml for the 1, 2 and for 3, 4 entries, respectively. CI, combination index (Chou, 2006).*

### Synergistic Effect of Terpenes

**FIGURE 3:**
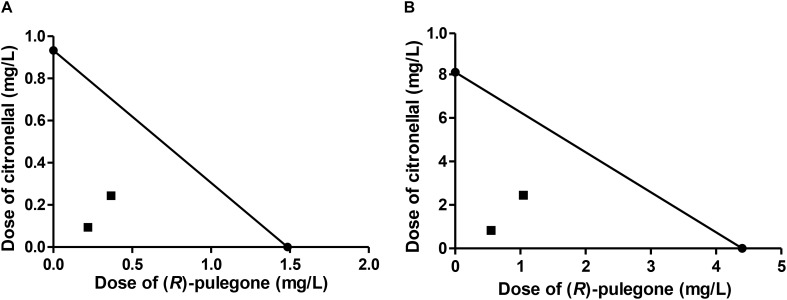
Isobologram corresponding to the (*R*)-pulegone-citronellal mixtures as fumigants against *M. domestica*. The MM was considered with **(A)** (*R*)-pulegone or **(B)** citronellal as substrate of P450. The isobolograms suggest a synergic effect in each case.

Our third case of study, the mixture of (*R*)-pulegone and citronellal, showed quite different results from those predicted by MM (Table 6), with observed LC_50_ values being markedly lower than expected. Moreover, CI values were quite far below one, suggesting a real synergism between these two terpenes, which was supported also by the isobolograms (Figure 3). Therefore, we used a convenient tool for the study of synergism by calculating the DRI (Chou, 2006), that estimated how much the dose of each compound in the synergistic combination may be reduced compared with the doses of each compound alone. According to DRI values, the (*R*)-pulegone/citronellal combination allowed for a reduction of up to 8 times the dose of (*R*)-pulegone and up to 10 times the dose of citronellal, thus showing high effectiveness and strong synergic interaction for this combination, possibly at the site of action (Table 6).

**Table 6:** Fumigant toxicity of (*R*)-pulegone and citronellal mixtures against *Musca domestica* adults.

Entry	Combination (% w/w)	Observed^a^ LC_50_ mg/L	Predicted (MM)^b^ LC_50_ mg/L	CI	DRI
1	(*R*)-pulegone	0.26 (0.197–0.34)	1.29	0.25	7
	citronellal (70:30)				10
2	(*R*)-pulegone	0.72 (0.22–1.19)	1.23	0.61	3
	citronellal (60:40)				3
3	(*R*)-pulegone	1.38 (0.70–2.12)	6.06	0.22	8
	citronellal (40:60)				10
4	(*R*)-pulegone	3.49 (1.36–5.43)	6.46	0.27	8
	citronellal (30:70)				7

*^a^Values in brackets are the 95% confidence interval. ^b^For calculation of LC_50_ (MM), values used for (R)-pulegone were 1.7 and 4.4 mg/L for the 1, 2 and for 3, 4 entries, respectively. Values used for citronellal were 0.9 and 8.1 mg/ml for the 1, 2 and for 3, 4 entries, respectively. CI, combination index and DRI: dose reduction index (Chou, 2006).*

### Determination of Metabolite of (*R*)-Pulegone/Citronellal Mixture

**FIGURE 4:**
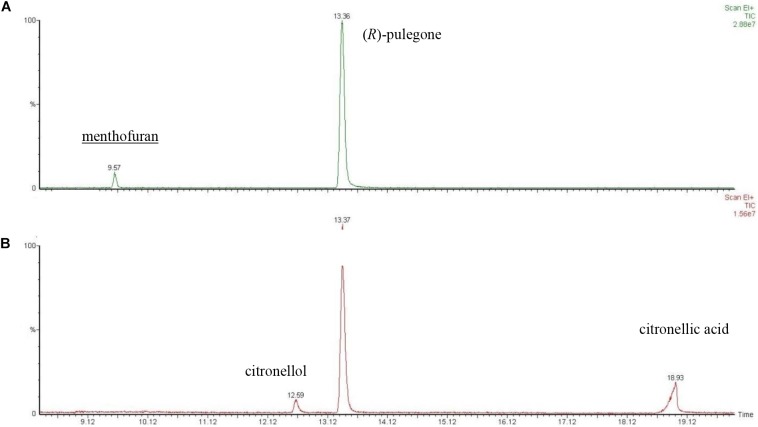
GC-MS chromatogram of headspace of the desorption (60°C) from dead flies previously fumigated with **(A)** (*R*)-pulegone/citronellal (70:30) and **(B)** (*R*)-pulegone/citronellal (30:70). Linear retention index (RI) and retention index (RI lit) taken from literature (Adams, 2007) were as follows: menthofuran 1160 and 1164; citronellol 1221 and 1226; (*R*)-pulegone 1232 and 1237; citronellic acid 1315 and 1312, respectively.

Due to the low LC_50_ values observed for the (*R*)-pulegone/citronellal mixture, the metabolic contribution to such values could not be inferred from their comparison. To deal with this problem, we explored the metabolic response of *M. domestica* to these mixtures at molecular level. It is known that flies fumigated with (*R*)-pulegone transform this compound into menthofuran (Rossi et al., 2012), but the metabolic product of citronellal was not known. Therefore, we first studied the metabolism of citronellal and determined that this terpene is converted to citronellic acid. Then, we determined the metabolites of a mixture dominated by (*R*)-pulegone [i.e., (*R*)-pulegone/citronellal 70:30], which yielded menthofuran but no detectable citronellic acid (Figure 4A), whereas in a citronellal rich mixture [i.e., (*R*)-pulegone/citronellal 30:70], we detected citronellic acid and citronellol but not menthofuran (Figure 4B). These results confirm our interpretation that the major terpene in a mixture is preferentially metabolized by the fly, while the minority component is not usually dealt with.

### Relevance of the Metabolic Model in the Context of Environmentally Friendly Pesticides

Essential oils have long been, and still are, extensively studied in the context of insecticidal action. This interest is fueled by their biodegradability, low or null toxicity to humans (they are frequently found in edible, medicinal and spice plants) and other characteristics that make them environmentally friendly. Many studies have tried to disentangle which terpenes, within the essential oil mixtures, are responsible for insecticidal activity. Although some have successfully done so (Lee et al., 2001), many studies failed to identify the active terpene (Bekele and Hassanali, 2001), because individually tested components tend to show less toxicity (greater LC_50_) than the essential oil containing them.

Our research has shed a new light on this problem, by showing that the toxicity of a given terpene, as measured by its LC_50_, includes amounts that do not actually reach the site of action because they are detoxified by the insect metabolic machinery. Moreover, we have devised and tested a new way of analyzing synergistic interactions, incorporating this metabolic response. By using this metabolic model, we have further shown that, in a mixture, a minor terpene may intoxicate the insect at a dose even lower than its specific LC_50_ because it is less targeted by the detoxification system, which is engaged in fighting the main component in the mixture. These findings provide an alternative mechanism for the higher toxicity of essential oils with respect to their components, and emphasize the need to integrate the insect metabolic response in the study of terpenes as potential insecticides. Taking into account that intoxication includes absorption, activation and detoxification, we cannot rule out the possibility that other variables, such as the volatility of the compounds, their chemical structure, physicochemical properties or selective penetration (in the case of mixtures) (Tak and Isman, 2015), may also be involved in the overall toxicity. Nonetheless, the close parity between the predicted LC_50_ by MM and the observed values strongly indicates, at least for the twelve combinations studied here, the dominant role of metabolic detoxification for terpene mixtures toxicity.

Since a significant synergistic relationship has been demonstrated in some terpenes, a more profound understanding of such relationships in a wider context may play an important role in the development of new botanical insecticides (Pavela, 2014). For example, a possible new avenue, prompted by our findings, could use substances that cause poor acute toxicity by themselves and incorporate them as minority components in a mixture, in order to significantly and even dramatically increase the effectiveness of other terpenes, as shown here for citronellal. In this way, a relatively inactive substance may actually play an important role in the intoxication of the target insect, and could thus be incorporated in the formulation of botanical insecticides, based either on pure terpenes or on essential oils containing the synergistic terpenes.

Finding important synergies is relevant for the future applicability of essential oils or terpenes as insecticides. Plants yield small amounts of essential oil (barely 0.1–3% of their biomass), therefore these compounds are scarce and expensive (Regnault-Roger et al., 2012; Attia et al., 2013). In addition, the doses needed to control insects are frequently above human recognition thresholds (0.1–4.0 ppm, dependent on the terpene) (Tamura et al., 1996), hindering their use and application, especially for domestic products. In the present work, we have found that combining terpenes in unequal proportions to avoid metabolization of the most toxic compound increases toxicity by 2–31 times compared to the corresponding individual terpenes. Such effective mixtures are more likely to be marketable.

Finally, we must point out that the metabolic contribution to LC_50_ has so far been addressed only for *M. domestica.* A similar mechanism is likely to occur in other insects, given that metabolic pathways tend to be shared by many species and that a wide range of insect P450s are able to inactivate these plant defensive compounds (Schuler, 2011). Further exploration of this possibility is highly important if we are to produce a general method with which to assess the best terpene combination for a range of pest species.

## Author Contributions

SP conceived and designed the experiments. ES and AB performed the experiments. MP and GF contributed analysis tools. SP, GV, MC, and MD wrote the paper.

## Conflict of Interest Statement

The authors declare that the research was conducted in the absence of any commercial or financial relationships that could be construed as a potential conflict of interest.
